# Reduced cortical GABA and glutamate in high schizotypy

**DOI:** 10.1007/s00213-021-05867-y

**Published:** 2021-06-19

**Authors:** Petya Kozhuharova, Andreea O. Diaconescu, Paul Allen

**Affiliations:** 1 Centre for Cognition, Neuroscience and Neuroimaging, Department of Psychology, University of Roehampton, Holybourne Ave, Roehampton, London, SW15 4JD UK; 2 Department of Psychiatry, Brain and Therapeutics, Krembil Centre for Neuroinformatics, CAMH, Toronto, Canada; 3 Department of Psychiatry, University of Toronto, Toronto, ON Canada; 4 Institute of Medical Sciences, University of Toronto, Toronto, ON Canada; 5 Department of Psychology, University of Toronto, Toronto, ON Canada; 6 Department of Psychosis Studies, Institute of Psychiatry, Psychology and Neuroscience, King’s College London, London, UK

**Keywords:** Glutamate, GABA, Spectroscopy, Schizotypy, Schizophrenia, Risk, Cortical, Metabolites

## Abstract

**Rationale:**

Abnormal functioning of the inhibitory gamma-aminobutyric acid (GABA) and excitatory (glutamate) systems is proposed to play a role in the development of schizophrenia spectrum disorder. Although results are mixed, previous 1H-magnetic resonance spectroscopy (MRS) studies in schizophrenia and clinical high-risk samples report these metabolites are altered in comparison to healthy controls. Currently, however, there are few studies of these metabolites in schizotypy samples, a personality dimension associated with the experience of schizophrenia and psychosis-like symptoms.

**Objectives:**

We investigated if GABA and glutamate metabolite concentrations are altered in people with high schizotypy. We also explored the relationship between resilience to stress, GABA metabolite concentrations and schizotypy.

**Methods:**

We used MRS to examine GABA and glutamate levels in the medial prefrontal cortex in people with low and high schizotypy traits as assessed with the Schizotypal Personality Questionnaire. Resilience to stress was assessed using the Connor-Davidson Resilience Scale.

**Results:**

Compared to individuals with low schizotypy traits, high schizotypy individuals showed lower cortical prefrontal GABA (*F* (1,38) = 5.18, *p* = 0.03, *η*^2^ = 0.09) and glutamate metabolite levels (*F* (1, 49) = 6.25, *p* = 0.02, *η*^2^ = 0.02). Furthermore, participants with high GABA and high resilience levels were significantly more likely to be in the low schizotypy group than participants with low GABA and high resilience or high GABA and low resilience (95% CI 1.07–1.34, *p* < .001).

**Conclusions:**

These findings demonstrate that subclinical schizotypal traits are associated with abnormal functioning of both inhibitory and excitatory systems and suggest that these transmitters are implicated in a personality trait believed to be on a continuum with psychosis.

**Supplementary Information:**

The online version contains supplementary material available at 10.1007/s00213-021-05867-y.

## Introduction

There is a growing consensus that psychosis exists on a continuum ranging from subclinical psychotic-like experiences in the general population to full-blown psychotic symptoms in clinical samples (Linscott and Van Os 2013). Psychotic-like experiences in healthy people, commonly referred to as schizotypy, represent a latent personality organization reflecting an underlying vulnerability to developing schizophrenia spectrum disorders (Barrantes-Vidal et al. 2014). Investigating schizotypal traits in non-clinical samples may provide information about neurobiological alterations underlying psychosis-like symptoms (phenotype) in the absence of confounding factors such as antipsychotic medication.

Schizotypal personality traits are qualitatively similar, but less severe than the symptoms seen in patients with schizophrenia. Relative to patients with schizophrenia, individuals scoring high on schizotypy trait measures show similar, albeit less severe, deficits in cognition and aberrations of perception, thought and beliefs. High schizotypy has also been linked with brain structural and functional abnormalities relative to control groups (Ettinger et al. 2014). However, fewer studies have investigated if there are neurochemical alterations in schizotypy populations similar to those reported in schizophrenia (Egerton et al. 2017) and predicted by animal models to influence psychosis symptomatology (Du and Grace 2013).

Whilst several animal studies show the significance of dopaminergic functions in psychosis risk/symptomatology (Du and Grace 2013; Grace 2010; Goto and Grace 2006), there have only been few studies examining GABAergic and/or glutamate function in schizotypy populations. The investigation of GABAergic and glutamatergic function is important because evidence from animal models and post-mortem studies of psychosis suggests that dysregulated excitatory and inhibitory neurotransmission plays an important role in the development of schizophrenia-like symptoms (Du and Grace 2013; Grace 2010; Goto and Grace 2006). The pre-clinical methylazoxymethanol acetate (MAM) rodent model of psychosis shows reduced parvalbumin expression in MAM-treated rats that may be linked to schizophrenia-like pathology (Gastambide et al. 2012). In particular, reduced parvalbumin expression may impact on prefrontal cortex (PFC) GABAergic interneurons that are known to be decreased in schizophrenia populations (Akbarian et al. 1995; Lewis et al. 2005). The MAM model also proposes dysfunction of the glutamatergic system as a possible mechanism that increases risk for psychosis (Marsman et al. 2013). The glutamatergic system is believed to affect synaptic plasticity and cortical microcircuitry, in particular (N-methyl-D-aspartate) NMDA receptor signalling (Merritt et al. 2016). Furthermore, the MAM model of psychosis emphasises a link between disrupted GABAergic and glutamatergic function and dysregulation of subcortical dopaminergic signalling (Grace 2016, 2010) that is thought to underlie positive symptoms such as delusions.

Consistent with pre-clinical work, studies in human subjects with a diagnosis of schizophrenia (Marsman et al. 2013; Merritt et al. 2016) report altered GABAergic and glutamatergic function relative to healthy control groups across a range of cortical and subcortical regions. However, a meta-analysis of 1H-MRS studies in schizophrenia and high-risk groups reports no overall difference in mPFC GABA metabolite concentrations relative to healthy controls (Egerton et al. 2017). Thus, it is unclear if mPFC GABA levels are altered in people with schizophrenia or in those with a high risk for the illness. Moreover, pharmacological challenge studies in humans report that the administration of NMDA receptor antagonists, such as ketamine and phencyclidine (PCP), induces symptoms that mimic the positive and negative symptoms seen in schizophrenia (Harrison and Weinberger 2005; Krystal et al. 1994; Moghaddam et al. 1997).

Crucially, however, studies investigating GABA and glutamate metabolite concentrations in high schizotypy samples are limited. Such studies are important if we are to better understand the role of (perturbed) inhibitory and excitatory neurotransmission across the psychosis continuum. One previous study investigating glutamate levels in individuals with high positive schizotypy traits reported no differences in metabolite concentration levels in the anterior cingulate cortex relative to a low positive schizotypy control group. There was however an interaction effect such that glutamate levels were negatively associated with the degree of cortical activation in response to emotional pictures in the striatum and the mPFC (Modinos et al. 2018). These preliminary findings suggest that cortical glutamate levels might be perturbed in high (positive) schizotypy in the context of affective function.

Given the paucity of 1H-magnetic resonance spectroscopy (MRS) studies in schizotypy populations, in the current study, we first sought to investigate GABA and glutamate metabolite concentrations in a sample of high schizotypy participants, relative to a low schizotypy control group. We used MRS with a voxel located in the medial PFC as pre-clinical models implicate this region in the neuropathology of psychosis and psychosis risk. Moreover, a number of previous MRS studies in CHR and schizophrenia samples have investigated this region and reported altered metabolite concentrations in the mPFC (Becker et al. 2003; Modinos et al. 2017; Mailly et al. 2013). Given that MRS studies in schizophrenia cohorts have reported both increased and decreased GABA and glutamate metabolite concentrations in prefrontal regions (Marsman et al. 2013; Merritt et al. 2016), we cannot predict a direction of this effect and instead predicted that relative to low schizotypy participants, high schizotypy participants will show *altered* GABA and glutamate metabolite concentration in the mPFC.

Our second aim was to explore if the relationship between cortical GABA metabolite concentrations and schizotypy was affected by resilience (to stress). Work in animals shows that stress, induced by injecting rats with corticosterone, leads to a decrease of mRNA for GAD67, the enzyme that synthesises GABA (Stone et al. 2001; Deslauriers et al. 2013; Giovanoli et al. 2013). Moreover, animal models of schizophrenia indicate that the GABA system is strongly influenced by environmental stress (Zhang et al. 2010; Guidotti et al. 2011), and that in humans, stress exposure in adolescence can lead to prodromal psychotic symptoms (Zimmerman et al. 2013). Taken together, these findings suggest that stress may affect GABAergic interneurons leading to the emergence of schizophrenia-like symptoms (Powell et al. 2012). However, to date, no study has investigated the relationship between stress/resilience, GABA metabolite concentrations, and schizotypy. To address this, we explored if the relationship between resilience levels and GABA metabolite concentrations differed across low and high schizotypy groups. We hypothesised that mPFC GABA metabolite concentrations would interact with resilience scores to predict SPQ group membership (high vs low), and that low and high schizotypy groups would show a different relationship between GABA metabolite concentrations and resilience levels.

## Methods

### Participants

One thousand three hundred forty-two participants responded to an online survey advertised via social media and were pre-screened using the Schizotypy Personality Questionnaire (SPQ; Raine 1991) and the Marlowe-Crowne Social Desirability Scale (SDS; Fischer and Fick 1993). All participants that took part in the MRI study were recruited from the student population of the Royal Holloway University of London. Exclusion criteria were defined as the presence of contraindicators for MRI scanning (presence of metal, etc.), current use of prescribed medication for neuropsychiatric disorders or history of neuropsychiatric disorders, current use or history of illicit substance misuse. These criteria were assessed via self-report and pre-screening for MRI scanning. The SDS questionnaire was used to exclude participants that give mainly socially desirable answers; thus, only subjects scoring 8 or higher on this measure were excluded to control for socially desirable responding (Fischer and Fick 1993).

Subjects were invited to take part in the study based on their SPQ score. The aim of the study was to recruit the bottom and top 10% (decile) of the schizotypy continuum (SPQ distribution); thus, individuals scoring below 12 and above 41 points on the SPQ were invited to take part (as informed by previous research; Raine 1991). The SPQ provides an overall measure of individual differences in schizotypal personality traits and can be reduced to three latent dimensions (positive, disorganised and negative; Raine 1991), mimicking the symptom clusters of schizophrenia and clinical high-risk states. The final sample included 27 participants in the high schizotypy group (HS; 17 females, age range 18–22, M = 19.25, SD = 1.05) and 26 participants in the low schizotypy group (LS; 19 females, age range 18–27, M = 20.38, SD = 2.02). The sample size was based on a generic effect size (0.6 to 0.8) calculation that would allow us to detect a medium effect based on 80% power and an alpha = 0.05 in our sample.

Ethical approval for the study was obtained from the University of Roehampton’s Ethics Committee and all participants provided informed written consent before initiating any study procedures. Participants were compensated for their time (£40 cash payment and a high-resolution anatomical scan of their brain).

Participants also completed demographic and substance use measures and the 25-item Connor-Davidson Resilience Scale (CD-RISC; Campbell-Sills and Stein 2007) to measure their resilience levels. The CD-RISC scale was developed to measure the ability to cope with adversity, with higher scores indicating greater resilience. Higher CD-RISC scores indicate higher levels of resilience and an increased ability to cope with stress (Campbell-Sills and Stein 2007). On the day of MRI scanning, participants completed a validated short version of the Wechsler abbreviated scale of intelligence (WASI II) to assess intellectual ability (IQ). We also used the Brief Symptom Inventory-Short Form (SF, Furlanetto et al. 2005) and Spielberger State Trait Anxiety Inventory (Grös et al. 2007) to measure depressive and anxiety traits in participants.

### MRI acquisition

All MRI scans were acquired on a 3 T Siemens Magnetom TIM Trio scanner using a 32-channel head coil. Structural T1-weighted magnetization-prepared rapid acquisition gradient echo (MP RAGE) images were acquired with a spatial resolution of 1 mm × 1 mm × 1 mm, in plane resolution of 256 × 256 × 176 continuous slices, TR of 1900s and scanning time of approximately 5 min. T1 MP RAGE scans were acquired for localization of the spectroscopy voxel placement and were subsequently segmented into tissue maps to allow volume correction for grey and white matter as well as CSF within the mPFC voxel, i.e. partial volume tissue contamination.

### 1H-MRS data acquisition and analysis

**Fig. 1:**
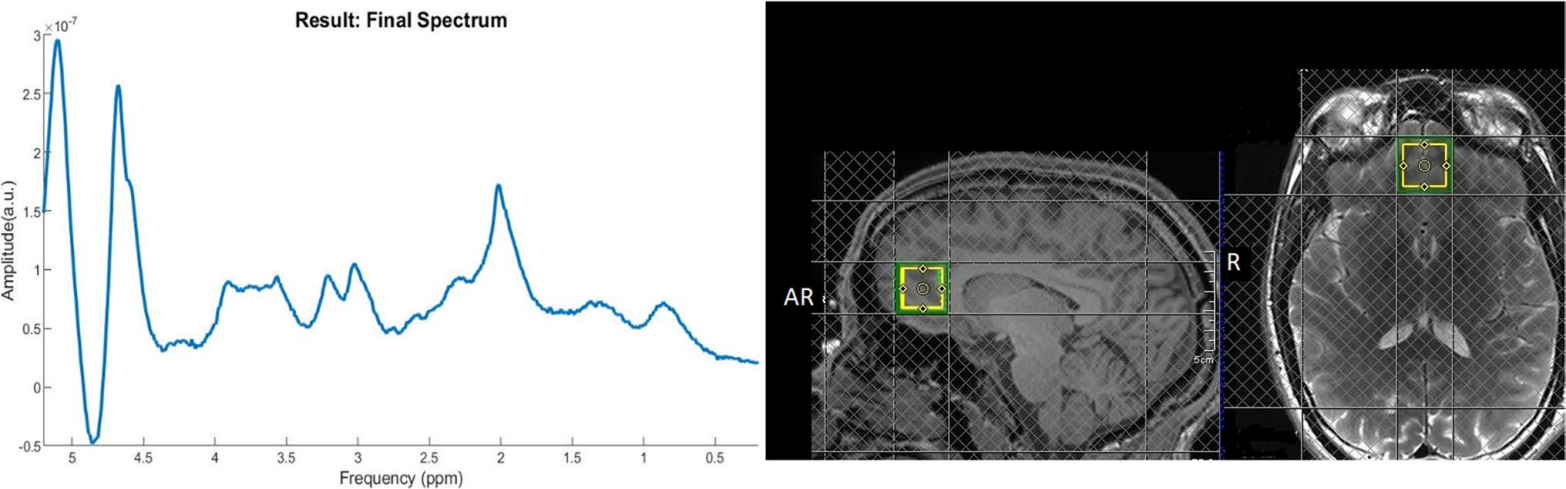
Voxel placement in the mPFC shown on an example participant (sagittal and axial orientation) and a representative 1H-MRS final spectra included from LCModel

1H-MRS in vivo spectra were acquired from a 20 × 20 × 20 mm voxel located in the bilateral medial PFC during rest (Fig. 1). The reasoning for choosing a medial PFC position was two-fold. First, lateral voxels can be harder to place due to tissue boundaries. Second, the medial PFC has shown abnormal GABA levels in schizophrenia patients in a number of previous studies and is one of the most commonly used MRS voxel placements for psychosis populations (Egerton et al. 2017; Modinos et al. 2017; Stone et al. 2009).

The voxel was positioned manually by reference to an axial T1-weighted gradient echo image (Fig. 1). Spectra were acquired using spin echo full intensity-acquired localized spectroscopy (SPECIAL; Mlynárik et al. 2006). 1H-MRS sequence was acquired with water suppression (TR 3000 ms, TE 8.5 ms, phase cycle auto, 192 averages from the right PFC voxel) in each participant (Godlewska et al. 2015). Water unsuppressed spectra (16 averages) were also acquired. Six outer volume suppression slabs were applied (one on each side at 5 mm from the edge of the cubic voxel) to suppress signals originating from outside the volume of interest and to minimize motion-related image-selected in vivo spectroscopy subtraction artefacts. Spectra were analysed using LCModel 6.3-1 N with the basis set consisting of 19 simulated basis spectra: alanine (Ala), ascorbate (Asc), aspartate (Asp), creatine (Cr), γ-aminobutyric acid (GABA), glucose (Glc), glutamine (Gln), glutamate (Glu), glycine (Gly), glutathione (GSH), glycerophosphocholine (GPC), phosphocholine (PCh), lactate (Lac), myo-inositol (mI), N-acetylaspartate (NAA), N-acetyl aspartate glutamate (NAAG), phosphorylethanolamine (PE), scyllo-inositol (Scyllo) and taurine (Tau).

The basis set was simulated using FID-A [61] for TE = 8.5 ms, magnetic field strength = 3 T and assuming ideal RF pulses. We excluded spectra with Cramer-Rao lower bounds (CRLB) > 20% as reported by LCModel. Line widths and signal-to-noise ratios were calculated by LCModel for both LS and HS groups (see Results). All spectra had a line width < 8 Hz and an SNR > 40 (Godlewska et al. 2015). Following these quality control checks, 8 participants from the HS group and 5 participants from the LS group were excluded due to CLRB ratios for GABA > 20%. Thus, the reported results for GABA are from 40 subjects in total (19 HS and 21 LS). For analysis of glutamate, quality control checks indicated that 2 subjects from the HS group had to be excluded due to CLRB ratios for Glu > 20%, resulting in a total sample of 25 HS and 26 LS individuals from the analysis of glutamate.

Metabolite levels have been shown to depend on the amount of cerebrospinal fluid (CSF), gray (GM) and white matter (WM) within the voxel (Srinivasn et al. 2006), and inter-individual differences in cortical gray matter (Huster et al. 2007). To account for these confounds, we used the T1-weighted anatomical images to estimate the gray and white matter content of the mPFC voxel in which the 1H-MRS measures were acquired using FSL FAST segmentation (Zhang et al. 2001). For every subject, tissue masks for CSF, GM and WM were created using FSL and the following procedure: registering the tissue template in MNI space to native space using FSL prior tissue probability maps, finding overlap between the tissue probability maps and the tissue template created in the previous step, applying a 0.4 threshold and binarizing the tissue templates, and finally generating the tissue masks by applying the prior in native space to the binarized tissue probability map.

Following this, for every individual subject, the placement of the MRS voxel was applied as a 20 × 20 × 20 mask (individually measured for each subject based on 1H-MRS acquisition) to the three respective tissue maps providing a segmentation quantity for each tissue in the specific 1H-MRS voxel placement. CSF, GM and WM were then accounted for in the expression of Glu and GABA levels using LCModel (Gasparovic et al. 2006); corrected metabolite levels are referred to as Glu-Corr and GABA-Corr using the formula Glu-Corr = (Glu*(43,300*GMV + 35,880*WMV + 55,556*CSF))/(35,880*(1 − CSF)) and GABA-Corr = (GABA*(43,300*GMV + 35,880*WMV + 55,556*CSF))/(35,880*(1 − CSF)).

To test for demographic differences between LS and HS groups, we used chi-square or independent sample *t*-tests. Differences between LS and HS groups in mPFC metabolite levels, as well as SNR, line width and CRLB, were established using multivariate analysis of variance (MANOVA) to control for multiple testing. These analyses also included BDI and STAI scores as covariates of no interest. Using logistic regression, we tested if resilience (CD-RISC scores) and GABA metabolite concentrations predicted SPQ group (low, high) membership. No assumptions of the logistic regression model were violated in the current samples. The analysis of multicollinearity revealed variance inflation factors of 3.42 and 4.71 for GABA_Corr and CD-RISC scores, respectively. For completeness, the regression model was repeated to investigate the effects of CD-RISC scores on glutamate metabolite concentrations and SPQ group. All analyses are conducted in R and reported at a significance level of *p* < 0.05. There were no a priori hypotheses for other 1H-MRS metabolite levels as our focus was on GABA and Glu metabolite concentrations due to their key role in neuropathology of schizophrenia and in psychosis risk (Egerton et al. 2014; Marsman et al. 2013). For completeness, NAA, myo-inositol and creatine (commonly reported metabolite concentrations) are reported in Supplementary Materials.

## Results

Due to the differing sample sizes for GABA and Glu metabolite concentrations resulting from quality control checks of the metabolite levels, we report the results separately. There was a significant positive strong correlation between the MRS measures of Glu and GABA in the participants in this study, *r* = 0.74, *p* < 0.01.

### MRS GABA metabolite concentrations

**Fig. 2:**
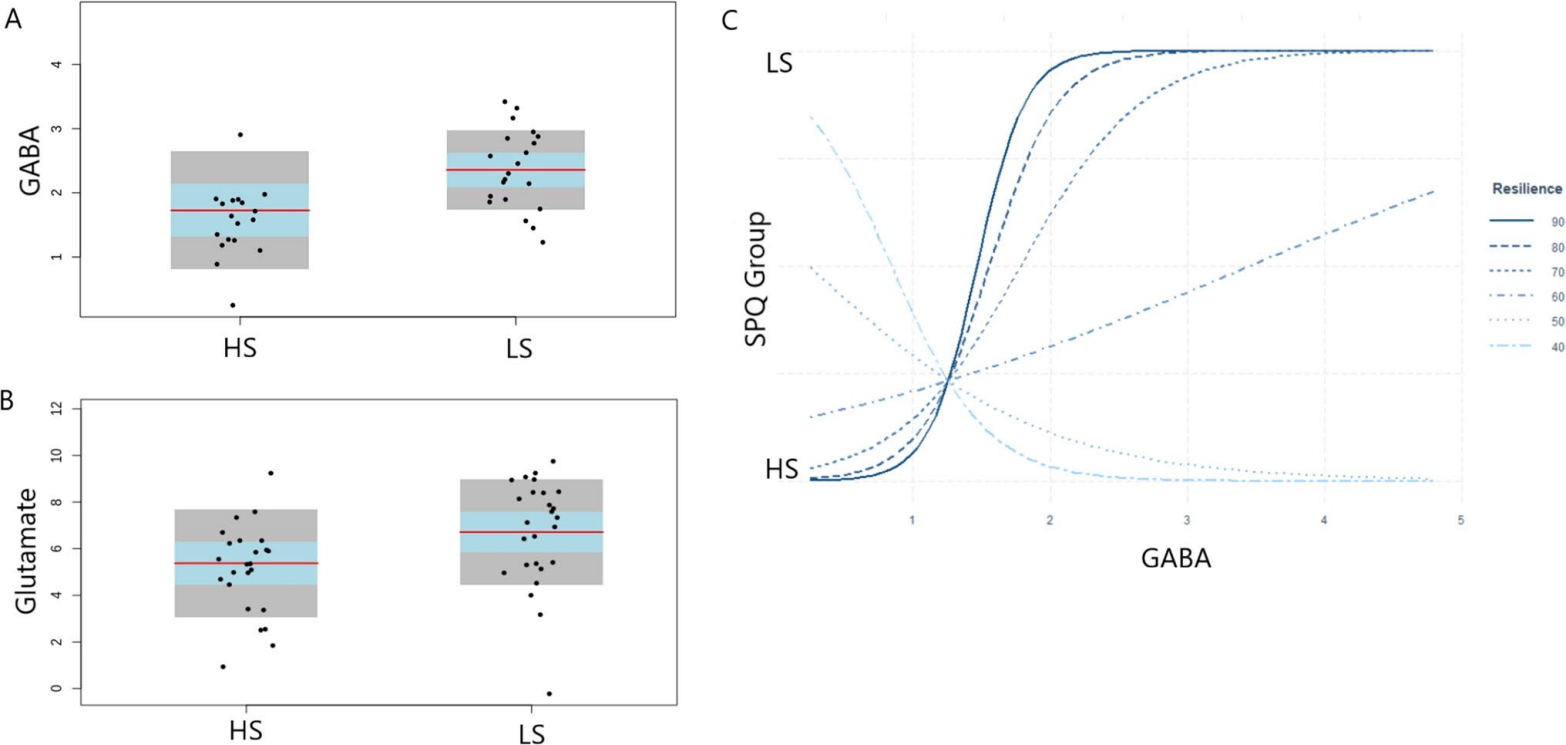
**a** GABA-corrected levels and **b** Glu-corrected levels by SPQ groups. Error bars show the standard error of the mean. **c** Logistic regression interaction effect with GABA_Corr levels on the x axis and SPQ group membership on the y axis; resilience is presented at the distributions of the resilience scores in the data

Table 1 summarises the sociodemographic sample characteristics for the analysis of GABA metabolite concentrations in LS and HS groups. HS and LS groups were matched for gender, age and IQ but differed on all schizotypy measures, as intended by design. The low and high schizotypy groups differ significantly on measures of depression and trait anxiety. ANOVA revealed that HS groups had significantly lower GM, WM and CSF tissue volumes in the mPFC voxel compared to LS. Summaries of the quality check data for the GABA set by group are presented in Table 2. No significant differences between groups were detected for SN ratio, line width or CRLB. Using corrected metabolite concentration values and including depression and anxiety scores as covariates of no interest, ANOVA showed that the HS group (M = 1.73, SD = 0.92) had significantly lower GABA-Corr levels than the LS group (M = 2.36, SD = 0.62), *F* (1,38) = 5.18, *p* = 0.03, *η*^2^ = 0.09 (Fig. 2a). Logistic regression showed that the interaction between GABA-Corr levels and CD-RISC scores (resilience) was a significant fit of the model, *χ*^2^(3) = 43.3, *p* < 0.04, Cox and Snell’s *R*^2^ = 0.42, Nagelkerke’s *R*^2^ = 0.73, and a significant predictor of SPQ group membership (*b* = 0.17, SE = 0.05, *z* = 3.08, *p* < 0.01). Figure 2 shows that participants with high GABA and resilience levels were significantly more likely to be in the low schizotypy group than those with low GABA and high resilience or high GABA and low resilience (95% CI 1.07–1.34).

**Table 1:** Demographic summary of questionnaire and tissue maps across the HS and LS groups for GABA metabolite analysis

Characteristic	LS (*n* = 21)	HS (*n* = 19)	F/*χ*^2^	*p*
Gender (male/female)	5/16	6/13	0.03	0.84
Age (years)	19.13 (SD = 2.10)	19.87(SD = 1.05)	1.05	0.31
IQ score	93.95 (SD = 9.13)	92.89 (SD = 9.69)	0.12	0.72
BDI-SF	4.04 (SD = 3.39)	12.44 (SD = 5.32)	6.89	< 0.01
STAI	66.92 (SD = 11.76)	101.25 (SD = 13.31)	9.93	< 0.01
SPQ total	6.86 (SD = 3.34)	45.8 (SD = 3.39)	133.7	< 0.01
SPQ cognitive perceptual factor	2.67 (SD = 2.13)	20.8 (SD = 3.96)	335.53	< 0.01
SPQ interpersonal factor	3.14 (SD = 2.26)	20.1 (SD = 3.81)	299.32	< 0.01
SPQ disorganised factor	1.67 (SD = 2.42)	10.7 (SD = 2.84)	118.86	< 0.01
Resilience	61.5 (SD = 13.7)	74.1 (SD = 13.0)	8.91	< 0.01
WM volume	0.36 (SD = 0.03)	0.27 (SD = 0.07)	27.15	< 0.01
GM volume	0.42 (SD = 0.03)	0.29 (SD = 0.11)	24.17	< 0.01
CSF volume	0.16 (SD = 0.02)	0.12 (SD = 0.03)	19.73	< 0.01

**Table 2:** Summary of quality measures for the GABA dataset and Glu dataset based on group and total sample

GABA	LS	HS	Total	Analysis (HS vs LS), F statistics
mPFC GABA	LS (*n* = 21)	HS (*n* = 19)	Total (*n* = 40)	
SN ratio	53.23 (SD = 9.98)	51 (SD = 5.24)	52.23 (SD = 7.82)	0.005, *p* = 0.34
Line width in Hz	6.68 (SD = 1.23)	5.76 (SD = 1.85)	6.27 (SD = 1.64)	0.006, *p* = 0.7
GABA CRLB (in %)	14.80 (SD = 3.17)	14.17 (SD = 2.50)	14.52 (SD = 2.88)	0.05, *p* = 0.49
Glu				
mPFC Glu	LS (*n* = 26)	HS (*n* = 25)	Total (*n* = 51)	
SN ratio	52 (SD = 9.04)	51.4 (SD = 6.64)	51.70 (SD = 7.88)	0.005, *p* = 0.78
Line width in Hz	6.76 (SD = 1.15)	5.97 (SD = 1.77)	6.20 (SD = 1.58)	0.006, p = 0.6
Glu CRLB (in %)	5.84 (SD = 1.97)	5.16 (SD = 1.65)	5.50 (SD = 1.83)	0.07, p = 0.18

### MRS Glu metabolite concentrations

Table 3 summarises the sociodemographic sample characteristics for the analysis of Glu metabolite concentrations in LS and HS groups. HS and LS groups were matched for gender, age and IQ but differed on all schizotypy measures, as intended by design. Similarly, to the results from the GABA analysis, HS groups showed significantly lower GM, WM and CSF tissue volumes compared to LS. Summaries of the quality check values for the Glu set based on group are presented in Table 2. Using glutamate-corrected metabolite concentration values and including STAI and BDI scores as covariates of no interest, ANOVA showed that the HS group (M = 5.38, SD = 2.32) had significantly lower Glu-Corr levels than the LS group (M = 6.71, SD = 2.28), *F* (1, 49) = 6.25, *p* = 0.02, *η*^2^ = 0.02 (Fig. 2b). Linear regression showed no significant interaction effects between glutamate levels and CD-RISC (resilience) scores.

**Table 3:** Demographic summary of questionnaire and tissue maps across the HS and LS groups

Characteristic	LS (*n* = 26)	HS (*n* = 25)	t/*χ*^2^	*p*
Gender (male/female)	7/19	8/17	0.05	0.93
Age (years)	19.25 (SD = 2.14)	19.94(SD = 1.35)	1.04	0.32
IQ score	95.1 (SD = 9.16)	93.8 (SD = 9.80)	0.26	0.61
BDI-SF	5.21 (SD = 4.15)	14.44 (SD = 5.10)	6.28	< 0.01
STAI	63.72 (SD = 12.16)	99.27 (SD = 11.13)	8.79	< 0.01
SPQ total	7.5 (SD = 3.65)	47.2 (SD = 5.28)	980.48	< 0.01
SPQ cognitive perceptual factor	2.69 (SD = 1.95)	20.7 (SD = 5.06)	284.21	< 0.01
SPQ interpersonal factor	3.65 (SD = 2.67)	21.3 (SD = 4.20)	324.13	< 0.01
SPQ disorganised factor	1.85 (SD = 2.31)	10.8 (SD = 2.69)	164.68	< 0.01
Resilience	73.58 (SD = 12.93)	58.36 (SD = 14.23)	15.93	< 0.01
WM volume	0.34 (SD = 0.07)	0.27 (SD = 0.07)	13.82	< 0.01
GM volume	0.41 (SD = 0.06)	0.29 (SD = 0.11)	20.03	< 0.01
CSF volume	0.16 (SD = 0.02)	0.12 (SD = 0.03)	20.91	< 0.01

## Discussion

Using 1H-MRS, the present study found that relative to the LS group, the HS group had significantly lower mPFC GABA and glutamate metabolite concentrations. These results are in line with previous studies in schizophrenia populations which show that patients have lower mPFC GABA levels compared to healthy controls (Marsman et al. 2013; Öngür et al. 2010; Zhilei et al. 2015; Rowland et al. 2013). It should be noted however that our results are in contrast to the overall body of evidence in schizophrenia and risk populations as meta-analysis has reported no differences between patients and healthy controls for mPFC GABA metabolite concentrations (see Egerton et al. (2017) for review). Studies investigating metabolite concentrations in individuals with a clinical high risk for psychosis, similarly to schizophrenia studies, have also found increased (Tayoshi et al. 2010), decreased (de la Fuente-Sandoval et al. 2016) or unaltered GABA levels in at-risk populations relative to healthy controls (Chen et al. 2014; Menschikov et al. 2016; Da Silva et al. 2019). Findings are further complicated by reports that in clinical high-risk individuals, mPFC GABA levels are negatively correlated with the severity of negative symptoms (Menschikov et al. 2016) and that unaffected siblings have significantly lower GABA levels compared with controls (Chen et al. 2014). One possible reason for differences found across different studies and study populations may be medication status, as unlike in schizophrenia patient sample, participants with high schizotypy and/or a clinical risk for psychosis will not usually have been exposed to antipsychotic medication. Moreover, unlike studies in psychosis risk populations (clinical/genetic), studies in schizotypy populations can provide important information about neurobiological mechanisms that underlie schizophrenia phenotype across the continuum, in non-help-seeking individuals.

Despite inconsistent findings in humans, animal models of schizophrenia indicate that dysfunction of the GABAergic neurotransmitter system plays a major role in the pathophysiology of schizophrenia (Du and Grace 2013; Grace 2010). The MAM model of psychosis suggests a link between disrupted cortical GABAergic function and the dysregulation of hippocampal dopaminergic signalling (Grace 2010, 2016). The model posits that dopaminergic hyperactivity is an indirect consequence of the reduced number of parvalbumin inhibitory interneurons in the hippocampus and the PFC (Lodge and Grace 2008; Zhang et al. 2002). Parvalbumin interneurons contain and release GABA that inhibits, or limits, the activity of the neurons that provide output from the hippocampus and prefrontal cortex (Grace 2010). Indeed, studies revealed that MAM-treated rats show a selective loss of parvalbumin-containing interneurons in both the hippocampus and the prefrontal cortex (Lodge et al. 2009). The mPFC can regulate hippocampal and subcortical dopamine neuron activity via the nucleus reuniens of the thalamus (Grace 2010). Thus, the current finding of reduced mPFC GABA levels in high schizotypy participants is broadly consistent with diminished GABAergic regulation from the mPFC shown in the MAM model of psychosis, but whether this is due to a reduced density of parvalbumin interneurons (as shown in the animal model) cannot be established using MRS.

We also found that the interaction between resilience scores (the ability to cope with stress) and mPFC GABA metabolite concentrations predicted schizotypy group (low, high). Participants with both higher resilience and higher mPFC GABA metabolite concentrations were significantly more likely to be in the low schizotypy group. Whilst this finding is not straightforward to interpret, it suggests that both high resilience and mPFC GABA levels, and how these variables interact, may protect individuals from psychotic-like experiences. This result is broadly consistent with pre-clinical models of psychosis that show that environmental stress affects the GABAergic system in humans (Zhang et al. 2010; Guidotti et al. 2011), consequently increasing risk for psychosis (Zimmerman et al. 2013). However, the association between resilience levels and mPFC GABA metabolite concentrations did not differ significantly in low and high schizotypy groups. Thus, the relationship between resilience and mPFC GABA levels, and how this differs in people who experience psychosis-like symptoms, remains unclear.

The results are consistent with models linking stress sensitivity with the experience of psychotic symptoms (Barrantes-Vidal et al. 2013). Previous work has shown that stressful situations and social stress are associated with momentary psychotic-like and paranoid symptoms for those high in positive schizotypy, but not those low in positive schizotypy (Barrantes-Vidal et al. 2013) and that the interaction between schizotypy traits and stress significantly predicted reasoning biases (Le et al. 2019). Studies have also shown that baseline cortisol levels in adolescents with schizotypal symptoms predict severity of their schizotypal symptoms later in life (Walker et al. 2010) suggesting further that stress interacts with schizotypal traits.

Cortical glutamate levels were also found to be significantly lower in high compared to low schizotypy individuals, a finding consistent with some previous studies in patients with schizophrenia (Marsman et al. 2013; Öngür et al. 2010; Goto et al. 2009). However, as with studies of GABA metabolite concentrations, increased cortical glutamate has also been reported in patients with schizophrenia (Choe et al. 1996; Rüsch et al. 2008; Moore et al. 2006). Mixed results are also reported in studies investigating clinical high-risk samples, where decreased (Natsubori et al. 2014) and increased cortical glutamate levels have been reported (Stone et al. 2009). Animal models of psychosis generally posit a role for increased glutamate levels, particularly in medial temporal lobe regions, that leads to reduced hippocampal volume (Lieberman et al. 2018) and increased subiculum output to the ventral striatum via glutamatergic pathways (Moore et al. 2006; Lodge and Grace 2011). However, decreased glutamate levels have been reported in the mPFC in MRS studies of patients with schizophrenia and CHR samples (Marsman et al. 2013), consistent with the current findings in high schizotypy individuals. The glutamatergic system is believed to affect synaptic plasticity and cortical microcircuitry, in particular NMDA receptor signalling (Harrison and Weinberger 2005). NMDA receptor antagonists, such as ketamine and phencyclidine (PCP) which reduce glutamatergic signalling, induce symptoms that mimic the positive and negative symptoms seen in schizophrenia (Adams and Moghaddam 1998; Krystal et al. 1994; Moghaddam et al. 1997). Injection of these NMDA receptor antagonists leads to decreased glutamate levels (Marsman et al. 2013; Moghaddam et al. 1997; Rowland et al. 2005) and animal studies show that the absence of NMDA receptor subunits can cause alterations at the molecular and behavioural level and produce schizophrenia-like symptoms (Marsman et al. 2013).

Changes in cortical inhibitory and excitatory signalling may result in a loss of synchronous cortical activity (Lisman et al. 2008; Lewis et al. 2012) and underlie the behavioural deficits commonly reported in schizophrenia and psychosis risk populations (Lisman et al. 2008; Lewis et al. 2012), such as emotional processing (Keefe et al. 2005) and social cognition (Kozhuharova et al. 2019). In terms of schizotypy as a personality dimension, alterations in cortical excitatory and inhibitory neurotransmission are likely to have implications for cognition, perception and interpersonal skills (which may form a continuum with the florid abnormalities seen in psychiatric disorders). Indeed, a recent systematic review conducted by our research group reported that high schizotypy (and clinical at-risk populations) show a tendency towards increased activity in frontal cortex during various emotional and social cognition tasks (Kozhuharova et al. 2019). There is also an established literature reporting behavioural and functional alterations in cognition/executive function and the underlying neural substrate in people with high schizotypal traits (Ettinger et al. 2015; Nelson et al. 2013; Kwapil and Barrantes-Vidal 2015). The current findings therefore have implications for cognitive and behavioural performance associated with high schizotypal traits. Animal model studies show that a functional loss of GABA-mediated inhibition diminishes gamma oscillations (Lodge et al. 2009) and impairs cognitive function (Enomoto et al. 2011; Gruber et al. 2010). There is also preliminary evidence that glutamate also affects cognitive performance (Ohrmann et al. 2007; Bustillo et al. 2011; Shirayama et al. 2010; Merritt et al. 2013). Schizotypy traits are also associated with impaired cognitive functioning (Cochrane and Reynolds 2012; Bedwell et al. 2009; Völter et al. 2012) and the current findings suggest that abnormal inhibitory and excitatory metabolite levels might be a mechanism that affects cognitive performance across the psychosis continuum.

Although much more work is needed to investigate the relationship between cortical GABA/glutamate signalling and behavioural measures, one previous study investigating glutamate levels in individuals with high positive schizotypy reports that glutamate levels were negatively associated with the degree of activation to emotional pictures in the striatum and the mPFC (Modinos et al. 2017). Although we acquired no behavioural measures here, future work should examine the relationship between GABA/glutamate and cognitive function, affective processing and emotion regulation in high schizotypy samples.

A limitation of 1H-MRS is that it measures total GABA concentrations within a relatively large voxel, which is determined a priori, and cannot discriminate between GABA levels in different cell types. This limits the application of 1H-MRS in addressing the cell- and network-specific GABA abnormalities hypothesised to occur in schizophrenia and psychosis risk (Lewis et al. 2012). For this reason, the current findings cannot inform on the specific mechanisms that might lead to reduced GABA and glutamate levels and we cannot test whether these results are due to reduced GAD67 or reduced density of parvalbumin interneurons, as suggested by animal models. The 1H-MRS GABA signal may reflect the entire GABA content of the voxel (that is, intracellular and extracellular, and involved in metabolism or neurotransmission). Recent work argues that the 1H-MRS GABA signal predominantly relates to extracellular, extra-synaptic GABA providing tonic inhibitory tone, rather than GABA involved in phasic synaptic neurotransmission (Stagg 2014). Theoretically, the 1H-MRS GABA signal should therefore be sensitive to GAD67 reduction, but we could not test this in the current study. Pharmacologically induced alterations in synaptic GABA may be more sensitively imaged with positron emission tomography (PET, Egerton et al. 2017). In future, combination of this approach with 1H-MRS in the same subjects, and potentially during the same scanning session on combined PET-magnetic resonance platforms, might investigate dysfunction of synaptic versus non-synaptic GABA and glutamate in schizophrenia.

Whilst mood and anxiety disorders can affect neurometabolite levels, and the low and high schizotypy groups did differ significantly on measures of depression and trait anxiety, significant group differences for both GABA and glutamate metabolite concentrations were observed after STAI and BDI scores were added as covariates of no interest. Affective symptoms are however common in people with schizophrenia (Huppert et al. 2001), clinical high risk (Fusar-poli et al. 2014) and in high schizotypy samples (Lewandowski et al. 2006), and are considered to be part of the schizophrenia phenotype (Van Os and Reininghaus 2016; Upthegrove et al. 2017). In practice, the distinction between schizotypal/schizophrenia symptomatology and comorbid depressive/anxious traits is difficult to establish and there is little evidence to instruct diagnostic separation (Malhi et al. 2008). A further limitation of the study is that we did not record any family history of neuropsychiatric disorders and, although self-report data was sought and presented (see Supplementary Material section), we did not conduct urine drug screening to objectively test recent substance abuse. However, the latter is not a common practice in MRI schizotypy studies (Modinos et al. 2010; Ettinger et al. 2012; Wang et al. 2015). Moreover, whilst analyses covaried for anxiety and depression scores, we were unable to conduct a full clinical assessment in our high and low schizotypy groups. Thus, it was not possible to delineate whether schizotypal symptoms, particularly negative and interpersonal symptoms, were primary to a mood and/or anxiety disorder. Future studies should conduct a detailed clinical assessment to better characterise symptom profiles in high schizotypy participants.

A final limitation of the sample is that only the SPQ total score was used to define groups and future work should aim to delineate the MRS correlates of the specific schizotypal subfactors further. Indeed, much of the work in experimental animals discussed earlier pertains to psychosis and psychotic-like symptomology, rather than the schizophrenia syndrome. However, our approach follows the design of many clinical studies in schizophrenia patients, who experience a constellation of negative, positive and cognitive symptoms. Future work should use updated versions of the SPQ with a Likert scale rather than a dichotomous (true/false) response. This would allow for a better measure of trait severity.

In conclusion, the current study utilised MRS methods to investigate GABA and glutamate levels in individuals with high and low schizotypy levels. In line with predictions from animal and post-mortem studies of schizophrenia, the current study is the first to report reduced levels of both GABA and glutamate metabolite concentrations in high schizotypy individuals compared to low schizotypy. Furthermore, the current work supports the role of stress sensitivity in the development of schizophrenia-like symptoms. Whilst our results suggest that individuals with high schizotypal personality traits show similar changes in mPFC GABA and glutamate metabolite concentrations to those reported in some studies in schizophrenia and psychosis risk cohorts, they are in contrast to meta-analysis findings in these groups. These findings also suggest that changes in prefrontal GABA levels may be related to stress sensitivity, although more work is needed to better understand the role of stress in this relationship. Whilst the study design does not allow us to draw any conclusions about risk factors for schizophrenia, the findings provide support for the notion of a neurobiological psychosis continuum, and for the role of stress sensitivity in the development of schizophrenia-like symptoms.

## Supplementary Information

Below is the link to the electronic supplementary material.

Supplementary file1 (DOCX 21 KB)
