# Supplementary material for: Reduced cortical GABA and glutamate in high schizotypy
Source: Psychopharmacology (Berl). 2021 Jun 19;238(9):2459–70. doi: 10.1007/s00213-021-05867-y (PMC8373725; doi:10.1007/s00213-021-05867-y)
Supplement: Supplementary file 1 — Supplementary file1 (DOCX 21 KB) [file 213_2021_5867_MOESM1_ESM.docx]

**Reduced cortical GABA and glutamate in high schizotypy**

Petya Kozhuharova^1^*, Andreea Diaconescu ^2^, Paul Allen ^1,3^

^1^  Centre for Cognition, Neuroscience and Neuroimaging, Department of Psychology, University of Roehampton, United Kingdom, [kozhuhap@roehampton.ac.uk](mailto:kozhuhap@roehampton.ac.uk)

^2^ Department of Psychiatry, Brain and Therapeutics, Krembil Centre for Neuroinformatics, CAMH

^3^ Department of Psychosis Studies, Institute of Psychiatry, Psychology and Neuroscience, King’s College London, London, United Kingdom

* Corresponding author

Email: [kozhuhap@roehampton,ac.uk](mailto:kozhuhap@roehampton,ac.uk)

Address: Department of Psychology, Holybourne Ave, Roehampton, London, SW15 4JD

Supplementary Material

We report analyses for three additional commonly used metabolites, namely Creatine (CR), Myo-inositol and N-acetylaspartate (NAA). CSF, GM and WM were then accounted for in the expression of these metabolite levels using LCModel (Morgenroth, et al., 2019) ; corrected metabolite levels have the affix Corr. These three metabolites were corrected using the following formula Corr = (metabolite *(43300*GMV + 35880*WMV + 55556*CSF))/(35880*(1−CSF)) (Morgenroth, et al., 2019).

Differences between LS and HS groups in mPFC metabolite levels, as well as SNR, Line Width and CRLB were established using multivariate analysis of variance (MANOVA) to control for multiple testing. Summaries of the quality check data for these metabolites are presented in Table S1. The final samples included in these analyses varied following exclusion of participants due to quality control checks. For Cr, quality control checks indicated that 1 subject from the HS group had to be excluded due to CLRB ratios for Glu > 20%, resulting in a total sample of 26 HS and 26 LS individuals. For the analysis of Myo-inositol, 6 participants from the LS group and 2 participants from the HS group had to be excluded due to CLRB rations > 20%, resulting in a total sample of 20 LS and 25 HS subjects. There were no excluded participants for the NAA analysis, resulting in a total sample of 26 LS and 27 HS subjects. No significant differences between groups were detected for SN ratio, Line width or CRLB for any of these metabolites.

ANOVA showed that the HS group (M = 3.27, SD = 3.03) had significantly lower CR Corr levels than the LS group (M = 5.2, SD = 1.78), F (1,50) = -4.43, p = 0.01, η2= 0.7. For Myo-inositol, the HS group (M = 2.37, SD = 3.33) did not differ from the LS group (M = 3.11, SD = 5.21), F (1, 43) = -1.29, p = 0.2. For NAA, the HS group (M = 4.16, SD = 6.27) had significantly lower NAA Corr levels than the LS group (M = 7.06, SD = 3.28), F (1, 51) = -4.73, p = 0.01, η2= 0.6.

Table S1. Summary of quality measures for the Cr, Myo-inositol and NAA dataset based on group and total sample.

| **Cr** | **LS** | **HS** | **Total** | **Analysis (HS vs LS), F statistics** |
| --- | --- | --- | --- | --- |
| mPFC Cr | LS (n = 26) | HS (n = 26) | Total (n = 52) |  |
| SN Ratio | 53.2 (SD = 9.91) | 51 (SD = 5.27) | 51.8 (SD = 7.80) | 0.005, p = 0.61 |
| Line width in Hz | 6.68 (SD = 1.03) | 5.96 (SD = 1.45) | 6.25 (SD = 1.64) | 0.006, p = 0.7 |
| Cr CRLB (in %) | 3.56 (SD = 1.08) | 3.72 (SD = 1.34) | 3.64 (SD = 1.21) | 0.8, p = 0.4 |
| **Myo-inositol** |  |  |  |  |
| mPFC Myo-inositol | LS (n = 20) | HS (n = 25) | Total (n = 45) |  |
| SN Ratio | 50.2 (SD = 9.32) | 51.4 (SD = 6.50) | 51.8 (SD = 7.62) | 0.005, p = 0.64 |
| Line width in Hz | 6.76 (SD = 1.15) | 5.97 (SD = 1.77) | 6.38 (SD = 1.38) | 0.006, p = 0.6 |
| Myo-inositol CRLB (in %) | 8.61 (SD = 5.14) | 9.59 (SD = 5.46) | 9.15 (SD = 5.28) | 0.7, p = 0.5 |
|  |  |  |  |  |
| **NAA** |  |  |  |  |
| mPFC NAA | LS (n = 26) | HS (n = 27) | Total (n = 53) |  |
| SN Ratio | 53.1 (SD = 7.2) | 52.4 (SD = 5.4) | 52.7 (SD = 7.82) | 0.005, p = 0.4 |
| Line width in Hz | 6.68 (SD = 1.34) | 5.86 (SD = 1.62) | 6.27 (SD = 1.34) | 0.006, p = 0.7 |
| NAA CRLB (in %) | 3.84 (SD = 1.40) | 4.76 (SD = 2.65) | 4.31 (SD = 2.17) | 1.41, p = 0.2 |

Table S2. Summary of the tobacco use, alcohol use and cannabis use across the two groups.

|  | LS | HS | | Analysis (HS vs LS), t statistics |
| --- | --- | --- | --- | --- |
| Daily tobacco use (number of cigarettes) | 1.69 (SD = 3.02) | | 1.22 ( SD = 2.94) | t (51) = 0.99, p = 0.32 |
| Daily alcohol use (in units of alcohol) | 2.23 (SD = 0.90) | | 2.40 (SD = 1.48) | t (51) = 0.59, p = 0.56 |
| Cannabis use* | 1.61 (SD = 1.44) | | 1.26 (SD = 1.20) | t (df = 51), p = 0.26 |

* Self-report scale as follows: 0 = No use, 1 = Experimental use, has tried occasionally, 2 = Occasional use, has used small quantities from time to time, 3 = Moderate use, has used in small quantities regularly / or large amounts occasionally, 4 = Severe Use, has frequently used large quantities, often to intoxication/debilitation

Reference:

Morgenroth E, Orlov N, Lythgoe DJ, Stone JM, Barker H, Munro J, Eysenck M & Allen P. Altered relationship between prefrontal glutamate and activation during cognitive control in people with high trait anxiety. Cortex 2019;117:53-63.
